# Apple Grading Based on Multi-Dimensional View Processing and Deep Learning

**DOI:** 10.3390/foods12112117

**Published:** 2023-05-24

**Authors:** Wei Ji, Juncheng Wang, Bo Xu, Tong Zhang

**Affiliations:** School of Electrical and Information Engineering, Jiangsu University, Zhenjiang 212013, China; jiwei@ujs.edu.cn (W.J.); w18981051316@icloud.com (J.W.); 18896655476@163.com (T.Z.)

**Keywords:** apple grading, multi-dimensional view, Yolov5s, Swin Transformer

## Abstract

This research proposes an apple quality grading approach based on multi-dimensional view information processing using YOLOv5s network as the framework to rapidly and accurately perform the apple quality grading task. The Retinex algorithm is employed initially to finish picture improvement. Then, the YOLOv5s model, which is improved by adding ODConv dynamic convolution and GSConv convolution and VoVGSCSP lightweight backbone, is used to simultaneously complete the detection of apple surface defects and the identification and screening of fruit stem information, retaining only the side information of the apple multi-view. After that, the YOLOv5s network model-based approach for assessing apple quality is then developed. The introduction of the Swin Transformer module to the Resnet18 backbone increases the grading accuracy and brings the judgment closer to the global optimal solution. In this study, datasets were made using a total of 1244 apple images, each containing 8 to 10 apples. Training sets and test sets were randomly created and divided into 3:1. The experimental results demonstrated that in the multi-dimensional view information processing, the recognition accuracy of the designed fruit stem and surface defect recognition model reached 96.56% after 150 iteration training, the loss function value decreased to 0.03, the model parameter was only 6.78 M, and the detection rate was 32 frames/s. After 150 iteration training, the average grading accuracy of the quality grading model reached 94.46%, the loss function value decreased to 0.05, and the model parameter was only 3.78 M. The test findings indicate that the proposed strategy has a good application prospect in the apple grading task.

## 1. Introduction

Apple quality grading that is effective and precise can increase the economic benefits of apple commercialization and help apples remain competitive in the market. However, most of the fruit classification and grading technology is still based on manual grading and mechanical grading, with low horizontal automation. In order to achieve low-cost automatic grading, more scholars have carried out research on image processing based on deep learning. Hamid [1] realized seed image recognition and classification based on MoblieNetV2 architecture and successfully classified 14 different kinds of seeds, with an accuracy of 98% and 95%, respectively, in the training set and test set. Aggarwal [2] used convolutional neural network CNN to achieve accurate multi-label classification in multi-label recognition and classification tasks. Gulzar [3] improved the MobileNetV2 architecture, combined with transfer learning technology to solve the problem of insufficient training data, and it performed well in the classification and recognition of the final fruit dataset.

Although the deep learning method in the above research performs well in some image processing tasks, there are still numerous challenges in the task of apple quality grading based on apple images. In the actual detection, the information contained in a single view of an apple is limited, and only when the classification algorithm obtains more complete apple surface information can it obtain more accurate classification results. Therefore, it is necessary to use multi-dimensional information of an apple as the basis for classification judgment. Su [4] developed a 3D model of the target through a multi-dimensional 2D image set, which made the judgment and analysis of the target more comprehensive. Ma [5] combined convolutional neural networks (CNNs) and long short-term memory (LSTM) to pay more attention to the correlation between multi-dimensional views. Shi [6] used bidirectional LSTM to construct a spatial feature aggregation module to fuse and analyze the apple information captured by three cameras with different perspectives. These studies could effectively improve the classification accuracy, but different perspectives resulted in different information being displayed for the apple views that the camera acquired. The apple top view offers less useful information, and the stem and calyx will obstruct subsequent flaws and color detection. The apple side view offers the most information and is the most useful for apple grading. In addition, the apple surface reflection and uneven illumination will also have a negative impact on the detection grading. These issues are currently being studied by an increasing number of academics. For defect detection, Fan [7] used lightweight convolutional neural networks that can achieve a detection speed of 5 per second, but the detection accuracy was not high enough to accurately distinguish defects from fruit stem. Liang [8] used the improved YOLOv4 model to grade apples according to the number and area of defects and achieved promising grading accuracy, but grading was based only on the surface defects of the apple.

The issues with the research mentioned above can be summed up as follows: when achieving the detection rate requirements of the apple quality grading pipeline, the surface information obtained by the grading algorithm is not sufficient, and it is simple to obtain views with fruit stems and calyx that will interfere with the grading. The apple surface in the view reflection and uneven illumination will also have a negative effect on the grade. In addition, there are still some problems in the quality classification algorithm, such as the features on which the grading is based, which are relatively simple, and the classification accuracy is not high. Aiming at the above problems, based on a four-lane apple grading machine developed by our research group, an apple quality grading method based on multi-dimensional view information processing was designed in order to meet the requirements of the apple quality grading pipeline for detection speed and improve the grading accuracy as much as possible. The main contributions of this paper are as follows: Firstly, based on the multi-dimensional apple surface information obtained by the four-lane apple sorting machine, the YOLOv5s model was improved to complete the processing of multi-dimensional view information. To enhance the ability to extract apple surface features without adding to the model computational load, ODConv dynamic convolution was introduced to the YOLOv5s model backbone, and the model neck was designed using the lightweight GSConv and VoVGSCSP backbone. Then, an apple quality grading method combining the Swin Transformer (SwinT) module and Resnet18 backbone was proposed. The unique shift window self-attention mechanism of the SwinT module enhances the model's ability to integrate the context information of apple surface features, making the judgment result more approximate to the global optimal solution, thus improving the accuracy of quality grading.

## 2. Materials and Methods

### 2.1. Apple Quality Grading Equipment

**Figure 1:**
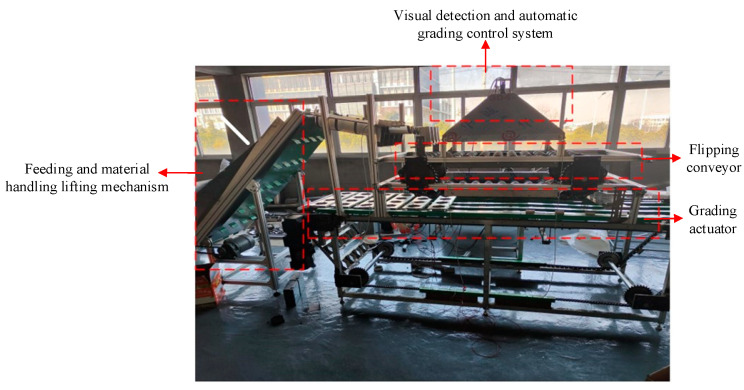
Four-lane apple detection equipment.

**Figure 2:**
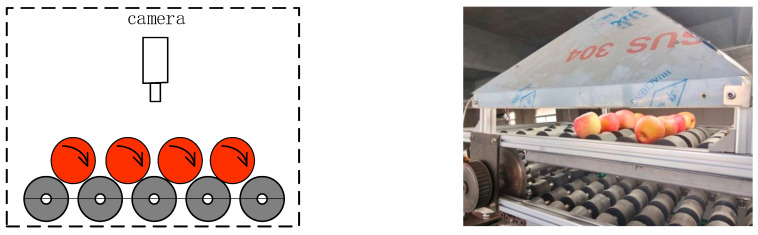
Roller turnover detection.

The four-lane apple quality grading equipment [9] developed by our research group, as shown in Figure 1, is mainly composed of a feeding lifting mechanism, flipping conveyor, visual detection grading control system, and grading actuator. The apples are transported to the flipping conveyor by the feeding lifting mechanism. The apples are flipped and rolled through the detection area on a flipping conveyor with a four-lane double conical roller bracket (Figure 2).

The visual inspection grading system uses an industrial digital camera with GigE interface (MER-531-20GC-P, 2592 × 2048) to obtain multi-dimensional information on apples and realizes quality grading of multiple apples at the same time through the grading method, and sends the grading results to the grading actuator. Finally, the apples of different grades are graded by the grading actuator.

### 2.2. Apple Quality Grading Standard

The visual inspections discussed in this paper were in accordance with the national standard (refer to the Chinese standard GB/T10651-2008 [10], Table 1) to carry out the quality grading.

### 2.3. Apple Quality Grading Method Design

The traditional apple grade detection based on machine vision is to put the apple on the belt of the belt conveyor and pass it through the detection area with a fixed attitude. The camera at a fixed position can only obtain information about a certain face of the apple; only using this as the basis for grading will significantly reduce the accuracy of grading. Aiming at the above problems, a flipping conveyor designed with a four-lane double conical roller bracket allows the apple to rotate freely and roll through the detection area. The camera fixed above the detection area can collect multi-dimensional information about the apple in real-time, which enables the grading to be based on more complete apple surface information.

**Figure 3:**
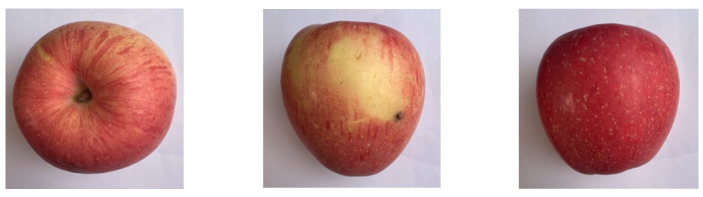
Multiple views of apple samples.

**Figure 4:**
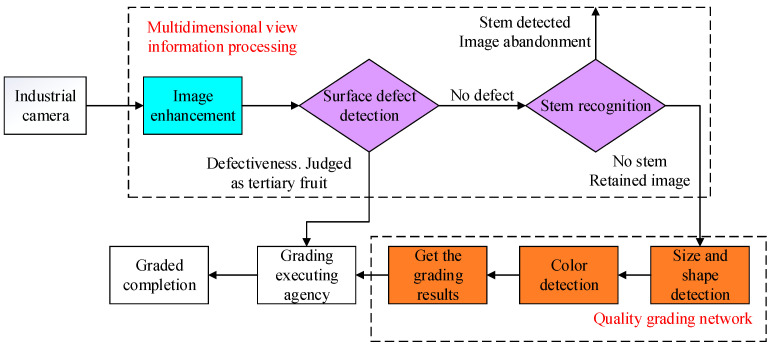
The overall process of quality grading.

However, the information contained in apple images of different sizes is different (Figure 3), and the side view can reflect the size, shape, color and other characteristic information of the apple. However, the top view will contain the stalk and calyx, which will interfere with the subsequent judgment of defect features and color features. Additionally, the quality of the grading algorithm itself will also directly affect the accuracy of the grading results. In view of the above problems, in this paper we designed an apple quality grading method based on multi-dimensional view information processing (Figure 4).

Firstly, the Retinex algorithm was used to enhance the apple image captured by the digital camera in real time to reduce the negative impact of the apple surface reflection and uneven illumination on the grading. Then, the improved YOLOv5s network model was used to identify apple surface defects. According to the surface defects of the apple, the quality of the apple is graded once. The apples with surface defects are directly determined as third-level fruits, while apples without surface defects continue to complete the process for recognition of fruit stems and calyx. The images with fruit stems and calyx are discarded, and only the side image data of apples are retained. Finally, the processed multi-view side information is saved and passed into the quality grading model in real time. The quality grading model makes comprehensive judgments and grades according to the size, shape, color and other characteristics of the apples to achieve accurate apple quality grading.

#### 2.3.1. Apple Multi-Dimensional View Information Processing

**Figure 5:**
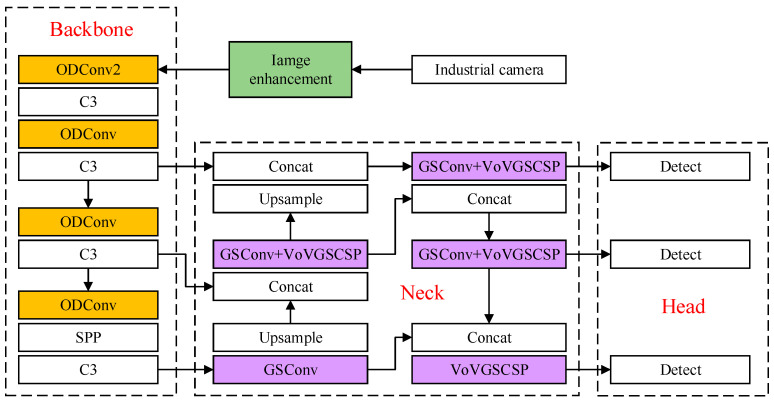
Processing with the multidimensional information view method.

The process of the multi-dimensional view information processing method designed in this paper is shown in Figure 5. Firstly, the Retinex algorithm is used to enhance the image, and then, the surface defect detection and recognition of fruit stem and calyx information are completed by the improved YOLOv5s model. We used ODConv dynamic convolution to replace the ordinary convolution in the model backbone to enhance the network’s ability to extract apple surface features. GSConv lightweight convolution is used in the neck part of the model to replace the ordinary convolution to receive feature mappings from the backbone of the model. Then, the VoVGSCSP lightweight backbone is used to promote the transmission of strong semantic features to complete the up-sampling and down-sampling of image features, and finally, the detection results are obtained by the detection head.

##### Apple Image Enhancement

Because the apple is a spherical fruit with large edge curvature, and the surface of apple skin itself has natural fruit wax, the surface of the apple easily reflects light, and the uneven illumination in the detection area will also affect the image quality. In order to reduce the influence of image noise on the grading, this paper uses the Retinex algorithm for image enhancement processing [11]. The image obtained by machine vision is composed of an incident image and reflection image, and the color of the apple surface will be determined by the ability of the apple to reflect light. $S\left(x,y\right)$ represents the apple image acquired by the camera, $R\left(x,y\right)$ represents the reflected image of the apple, $L\left(x,y\right)$ represents the incident image, and $\left(x,y\right)$ represents the coordinates of the pixels in the image. The relationship between these is as follows:

(1) $$R(x,y)=\frac{S(x,y)}{L(x,y)}$$

The logarithmic transformation of Equation (1) can be obtained as follows:

(2) logR(x,y)=logS(x,y)−logL(x,y)

Because the intensity of the incident light varies slowly on the apple surface, $L\left(x,y\right)$ can be represented by the low-frequency component in the apple image, and then the value of $R\left(x,y\right)$ can be calculated from Equation (2). The illumination change can be calculated and removed by calculating the weighted average $F\left(x,y\right)$ between the pixels in the original image and the surrounding area, and only the reflection attribute of the target is retained. Then, the incident image $L\left(x,y\right)$ can be expressed as follows:

(3) L(x,y)=F(x,y)×S(x,y)

**Figure 6:**
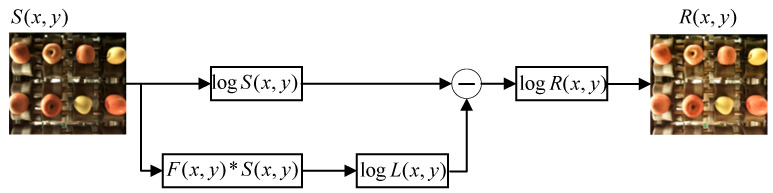
Overall process of the Retinex algorithm.

**Figure 7:**
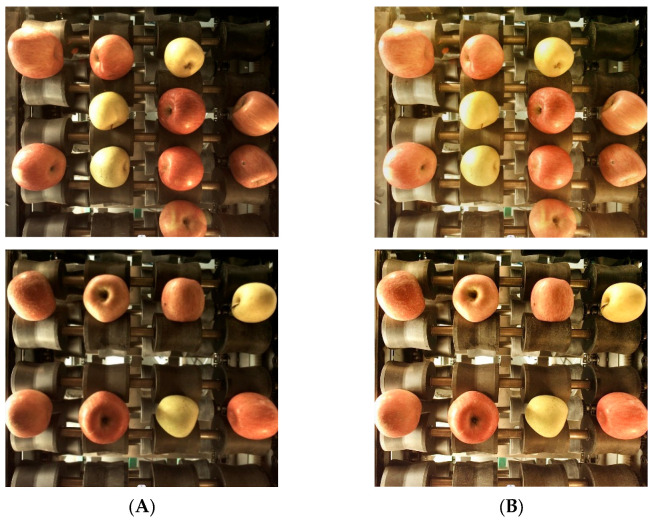
Images enhanced using the Retinex algorithm. (**A**) Original images;(**B**) Enhanced images.

Therefore, the reflection image $R\left(x,y\right)$ can be obtained from the machine vision image $S\left(x,y\right)$ by calculation, so as to achieve the purpose of filtering image noise. The flow of the Retinex image enhancement algorithm is shown in Figure 6. The comparison before and after image enhancement processing is shown in Figure 7.

##### Backbone Network Design

The accurate recognition of fruit stem, calyx, and surface defects requires that the recognition model have excellent nonlinear representation ability. In this paper, ODConv dynamic convolution [12] is used to replace the ordinary convolution in the backbone of YOLOv5s (Figure 5), so as to improve the ability of the network backbone to extract apple surface features. ODConv can be defined as follows:

(4) $$y=(\alpha_{w1}⊗\alpha_{f1}⊗\alpha_{c1}⊗\alpha_{s1}⊗W_{1}+\ldots +\alpha_{wn}⊗\alpha_{fn}⊗\alpha_{cn}⊗\alpha_{sn}⊗W_{n})\times x$$

where $\alpha_{wi}\in R$, $\alpha_{si}\in R^{k\times k}$, $\alpha_{ci}\in R^{C_{in}}$, $\alpha_{fi}\in R^{C_{out}}$ and represent the four attention scalars of the convolution kernel $W_{i}$, The convolution kernel $W_{i}$ is calculated along the convolution kernel number dimension, spatial dimension, input channel dimension, and output channel dimension of its kernel space, respectively. ⊗ represents multiplication operations along different dimensions of the kernel space. Compared with ordinary convolution, ODConv can simultaneously focus on the dynamics of four dimensions of kernel space (the number of convolution kernels, the spatial size of each convolution kernel, the number of input channels, and the number of output channels). Through the parallel strategy, a multi-dimensional attention mechanism is used to pay more flexible attention to the four dimensions of the kernel space.

**Figure 8:**
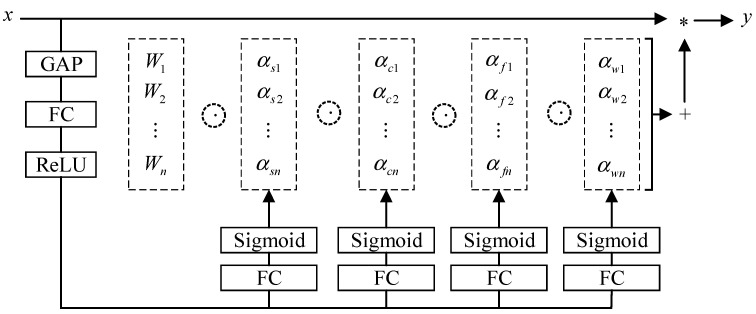
Structure of ODConv.

Specifically, the multi-head attention module is used to calculate the four kinds of attention $\alpha_{si}$, $\alpha_{ci}$, $\alpha_{fi}$ and $\alpha_{wi}$ of the convolution kernel. Its structure is shown in Figure 8. Firstly, the input is compressed into a feature vector of length $C_{in}$ by channel global average pooling (GAP). Subsequently, the fully connected (FC) layer and rectified linear unit (ReLU) are connected to the four branches, each branch with an FC layer. The output sizes are k×k, $C_{in}\times 1$, $C_{out}\times 1$ and n×1 respectively, and the Sigmoid function is used to generate the normalized attention scalars $\alpha_{si}$, $\alpha_{ci}$, $\alpha_{fi}$ and $\alpha_{wi}$ respectively. Finally, the calculation results of each branch are integrated to obtain the output of focusing on four dimensions of information, so as to better complete the image feature extraction and improve the recognition accuracy of the fruit stem, calyx, and surface defects of the model.

##### Network Neck Lightweight Design

**Figure 9:**
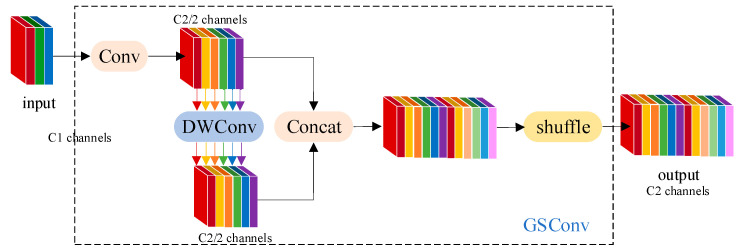
The structure of the GSConv module.

In order to improve the detection speed of apple grading, it is necessary to reduce the time for information processing. However, excessive reduction of model parameters will reduce the recognition accuracy of the model for apple surface features, so it is necessary to balance the detection speed and accuracy of the model. In this paper, the Neck part of the model is improved by lightweight [13], and the GSConv lightweight convolution is used to replace the ordinary convolution to receive the feature mappings from the model backbone. After the GSConv convolution, the VoVGSCSP lightweight backbone is used to complete the up-sampling and down-sampling of image features. The GSConv convolution structure is shown in Figure 9. GSConv divides the feature extraction operation to reduce the amount of model computation and uses a multi-channel dense convolution operation to preserve the hidden connections between each channel as much as possible to reduce the loss of semantic information. Finally, channel fusion was performed to complete feature extraction.

**Figure 10:**
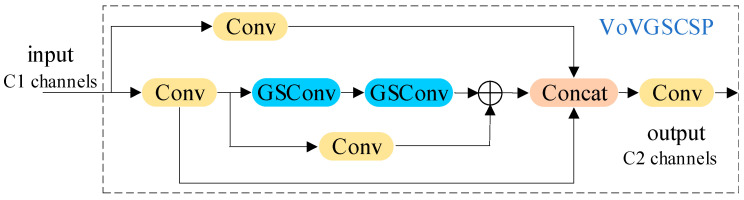
The structure of the VoVGSCSP module.

The VoVGSCSP backbone structure is shown in Figure 10. When the feature mappings reach the VoVGSCSP backbone through GSConv processing, it has become stable enough (the channel dimension reaches the maximum value, the width and height reach the minimum value), and there is less redundant repetitive information. The VoVGSCSP backbone uses two GSConv convolutions to quickly promote the transfer of strong semantic features, to complete up-sampling and down-sampling, and finally to reduce the model information processing time.

The lightweight improvement of the Neck part can avoid the insufficient expression ability of the model for the apple surface features as much as possible and reduce the overall computational cost of the model, give the network faster operating speed, reduce the information processing time, and better balance the detection speed and accuracy.

#### 2.3.2. Apple Quality Grading Model Design

The performance of the quality grading model itself will also directly affect the accuracy of grading results. In order to achieve the precise division of apple quality grades, this section will finish the scientific annotation of the apple dataset and design a high-performance quality grading algorithm.

##### Production of the Apple Grading Dataset

In this paper, YOLOv5s is used as the algorithm framework to build a quality classification model, and its grading accuracy is very dependent on scientific datasets. At present, many scholars have studied the automatic labeling technology of datasets. Mamat [14] has developed a simple and efficient automatic labeling technology of fruit images by using the YOLO model, and the accuracy of labeling fruit types can reach 99.5%. However, the research object of this paper is Red Fuji apple, which requires a standard grading dataset according to various characteristics of its surface. Therefore, 300 Red Fuji apples of different grades were randomly selected from Feng County, Xuzhou City, Jiangsu Province, China, and 1244 pictures were taken in the detection area of the four-lane apple grading equipment; according to the national standard GB/T10651-2008 (Table 1), the fruit diameter, shape, and color characteristics of the apple were extracted, and the scientific annotation of the dataset was completed.

**Figure 11:**
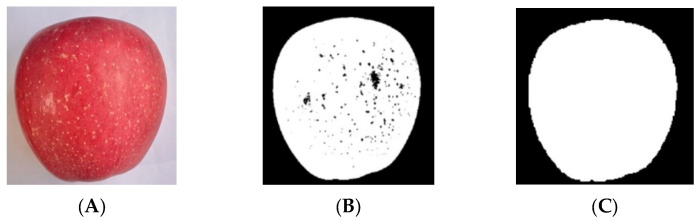
Image processing process. (**A**) Original image; (**B**) OTSU; (**C**) morphological denoising.

For the extraction of fruit diameter features, because the apple is a spherical fruit, it is difficult to determine the location of its spherical center, which leads to large errors in the direct measurement of fruit diameter size. Therefore, the size information of the apple is determined by calculating the area of the side image of the apple. The overall process is shown in Figure 11. Firstly, the OTSU threshold segmentation method [15] is used to segment the apple image and the background, and then morphological denoising is used to fill holes in the segmented image to eliminate noise. Finally, the apple diameter feature can be obtained by calculating the pixel area of the apple image.

For the extraction of fruit shape features, the radius corresponding to the area and perimeter of the standard circle is the same, and the difference between the two radii is larger when the circularity is worse. Therefore, the ratio of the radius corresponding to the area and perimeter of the apple is calculated to describe the shape characteristics of the apple. The radius corresponding to the area S is $r_{s}$, and the radius corresponding to the perimeter L is $r_{l}$.

(5) $$r_{s}^{2}=\frac{S}{\pi }$$

(6) $$r_{l}^{2}=\frac{L^{2}}{4\pi^{2}}$$

(7) $$e={\left(\frac{r_{s}}{r_{l}}\right)}^{2}=\frac{4\pi S}{L^{2}}$$

It can be seen from Equation (7) that when e is equal to 1, it is a standard circle, and the smaller e is, the worse is the circularity of the apple.

**Figure 12:**
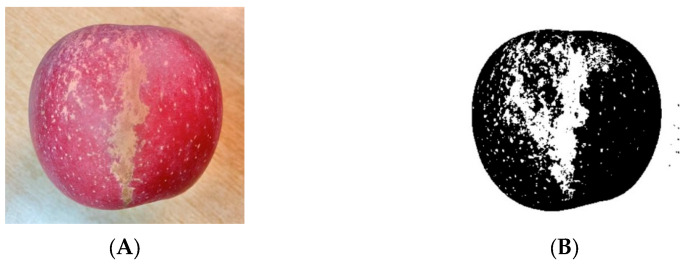
Color feature extraction. (**A**) Original image; (**B**) tinctorial yield 67.73%.

For the extraction of color features, the HSI color model is used to complete the extraction of apple color features. The feature extraction process is shown in Figure 12. The chromaticity in the range of 0~25 and 245~255 is selected as the bright red and thick red part of the apple surface color, and the ratio of the color to the total area of the apple is calculated as the apple color characteristic parameter.

The diameter, shape and color characteristics of apples were extracted, and the scientific labeling of the apple grade dataset was completed. A total of 1244 pictures, each containing 8 to 10 randomly graded apples, were divided into the training set and the test set in a ratio of 3:1. In the training set, there were 2698 targets of first-grade fruit, 2483 targets of second-grade fruit and 1743 targets of third-grade fruit.

##### Apple Grading Network Backbone Design

**Figure 13:**
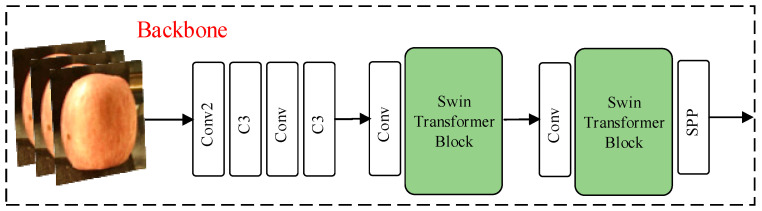
Structure diagram of the quality classification network backbone.

Accurate apple quality classification requires that the quality grading model have excellent feature extraction ability and be able to comprehensively analyze the global characteristics of apples. In this paper, the Swin Transformer (SwinT) [16] feature extraction module is added to stage 3 and stage 4 of the YOLOv5s Backbone (Figure 13), which increases the receptive field of the model for apple surface features and improves the quality grading accuracy.

**Figure 14:**
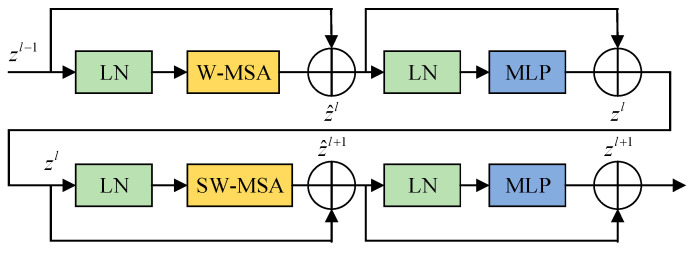
Structure of Swin Transformer blocks.

The structure of SwinT is shown in Figure 14, The input feature $z^{l-1}$ of window multi-head self attention (W-MSA) is passed through a Layer Norm (LN) layer, and then the output of W-MSA is added to obtain ${\hat{z}}^{l}$. ${\hat{z}}^{l}$ goes through another LN layer and the value obtained by the MLP and then adds to itself to obtain $z^{l}$. $z^{l}$ is then used as the input of shifted window multi-head self attention (SW-MSA), W-MSA is replaced by SW-MSA later, and other operations remain unchanged.

SwinT's unique shift window self-attention mechanism makes it more comprehensive than the commonly used C3 backbone to analyze the global effective information and obtain the global optimal solution. The shift window self-attention mechanism is mainly accomplished by the W-MSA operation and SW-MSA operation in the SwinT structure [17].

Window multi-head self attention (W-MSA) means that the image information is divided into windows, and then the transformer operation is performed on the image information in each window. The transformer operation can combine the features extracted from different regions of the window. The semantic information of the features in the window is obtained through comprehensive analysis and compared with the information processing of the whole image directly; the multi-window information processing can greatly reduce the computational complexity of the transformer operation.

Shifted window multi-head self attention (SW-MSA) enables the new window to contain not only part of the image information of the previous window but also part of the image information of the other window by different window cutting methods, and then performs the W-MSA operation on the image information in the new window. In this method, different feature mappings are obtained from W-MSA by different window cutting methods to realize the information interaction between windows.

SwinT is integrated into the backbone model to increase the model's ability to express itself comprehensively for a variety of features on the apple surface, bring the grading outcome closer to the global optimal solution, and improve the grading accuracy of the model. SwinT divides the image information into windows and then completes the information processing separately, which can effectively reduce the computational complexity and enhance the nonlinear expression ability of the model without excessively increasing the computational cost.

## 3. Results and Discussion

The algorithm program used in this paper was written in Python language on Pycharm and ported to an industrial PC with Windows 10 operating system and equipped with an Intel e5-2683 processor, NVIDIA GTX1080ti graphics card with 16 GB of video memory, and 64 GB of system memory. The effectiveness of the multi-dimensional view information processing method and the performance of the quality grading model were tested based on the developed four-lane apple grading machine.

In this paper, when testing the model performance, the indicators chosen to measure the rapidity were FPS, the number of model parameters and the total model size. FPS can reflect the speed at which images are processed. The number of parameters reflects the total number of parameters needed to run the model. The network structure determines the size of the model and reflects the space utilization of the network. The average precision AP was selected to measure the accuracy. AP is derived from the accuracy and recall rate.

### 3.1. Multi-Dimensional View Information Processing Method Test

#### 3.1.1. Ablation Experiment

In order to verify the effectiveness of the method designed for the apple grading task in this paper, ablation experiments were carried out on the fruit stem, calyx and surface defect recognition models (Table 2). In this paper, Red Fuji apples from Feng Xian County, Xuzhou City, Jiangsu Province, China, were selected for dataset production. Apples of different grades were randomly selected, and 1244 pictures were taken in the detection area of the four-lane apple grading device; each picture contained 8 to 10 target objects. LabelImg was used to label the stem and surface defects in the apple images. Pictures were divided into the training set and test set in a ratio of 3:1. The number of stem labels in the training set was 6803, and the number of surface defect labels was 943. The image category and target rectangle were saved according to the PASCAL VOC dataset format, and the annotation file was generated in XML format. Apples with large defect areas were discarded in the picking stage [18] without quality classification. While apples with surface defects were directly judged as third-grade fruits, surface defects were not further divided, and all shape defects were classified into one class. 

The recognition model for the fruit stem, calyx and surface defects is based on YOLOv5s, and the Retinex algorithm was added to enhance the input image. The common convolution was replaced by ODConv convolution in the backbone, and the GSConv convolution and VoVGSCSP lightweight backbone were introduced to improve the overall light weight of the neck. Without improvement, the detection accuracy of YOLOv5s for stem and surface defects was only 89.67%, and the number of model parameters was 7.4 M [19,20]. After adding the Retinex algorithm, the influence of illumination on apple surface features could be effectively reduced, and the model detection accuracy was improved by 4.65%. After adding ODConv convolution, the network backbone could enhance the extraction ability of apple surface features, and the detection accuracy of the model was improved by 2.64%, but the number of model parameters was increased by 1.92 M [21]. After the overall lightweight improvement of the neck, the model detection accuracy was 96.56%; only 0.42% of the detection accuracy was sacrificed, but the number of model parameters was reduced by 2.54 M, which optimized the model structure. This made the model lighter and faster.

#### 3.1.2. Comparative Experimental Results for Different Detection Models

**Figure 15:**
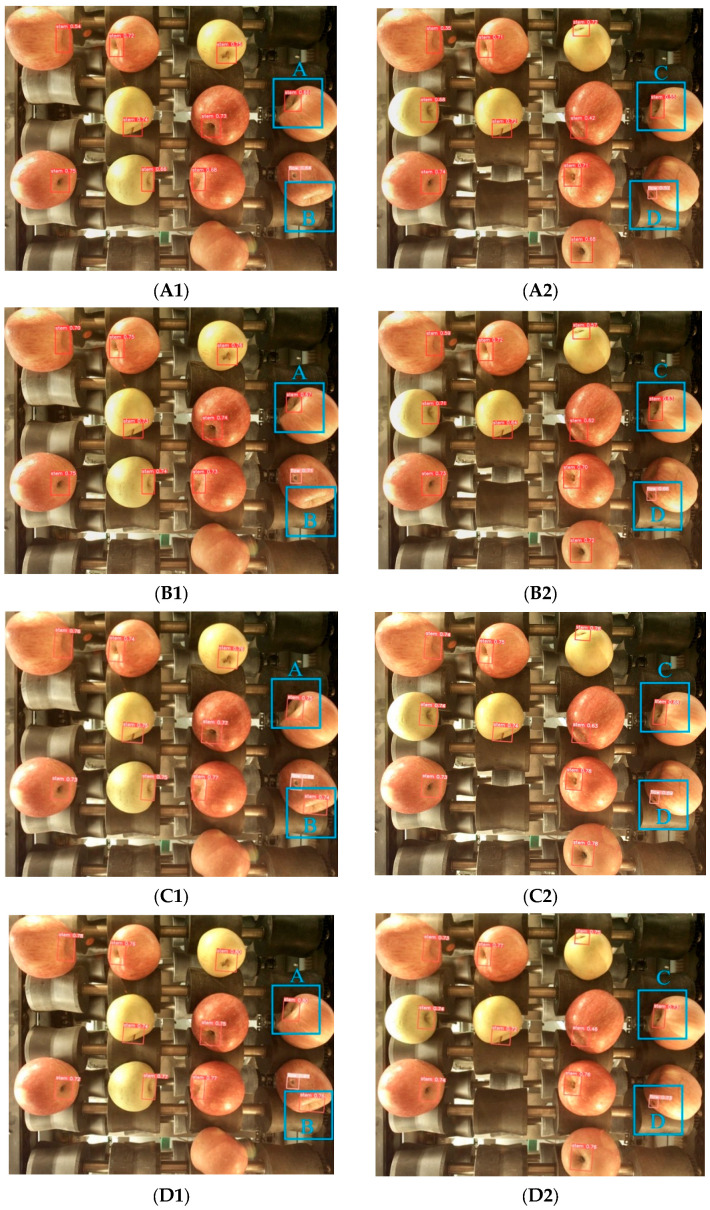
Stem and defect test results. (**A1**–**A2**) YOLOX-Tiny; (**B1**–**B2**) MobileNetV2; (**C1**–**C2**) ShuffV2-YOLOX; (**D1**–**D2**) Proposed method. (Blue box A, B, C) Stem detection at image edge positions; (Blue box D) Defect detection at image edge positions.

**Figure 16:**
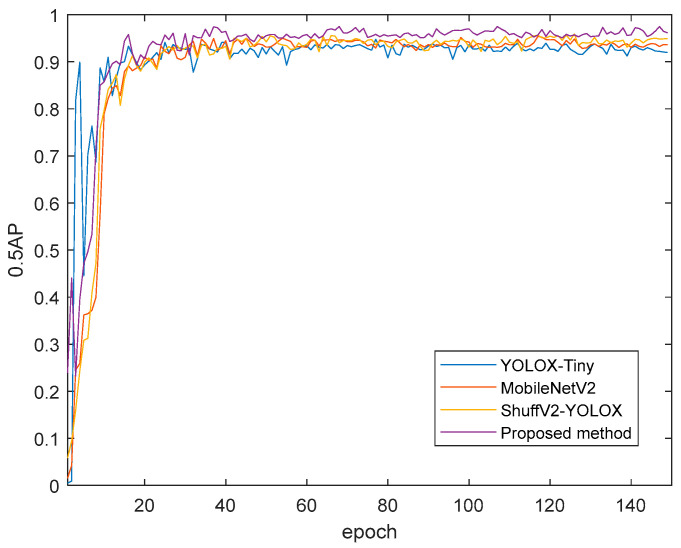
AP (0.5) Test results for different models.

In order to further verify the effectiveness of the proposed method in the apple grading task, the proposed model was compared with some mainstream detection models: YOLOX-Tiny [22,23], MobileNetV2 [24] and ShufflenetV2-YOLOX [25]. The experimental results in Figure 15 and Table 3 indicate the detection effect and performance data for different models in the recognition task. From the actual detection results in Figure 15, it can be seen that for the fruit stems in region B of the picture, YOLOX-tiny and MobileNetV2 did not recognize, while the recognition model studied in this paper accurately recognized the fruit stem information in region B, and the recognition accuracy was also better than that of the ShufflenetV2-YOLOX model. For the detection of fruit stems and surface defects at the shooting edge in picture regions A, C and D, the recognition accuracy of the other models was lower than that of the model studied in this paper. It can be seen from Table 3 that the recognition model in this paper has obvious advantages in detection accuracy and speed compared with YOLOX-Tiny and MobileNetV2. Compared with the ShuffenetV2-YOLOX model, although it had the fastest detection speed, the detection accuracy was not high enough. The comprehensive results show that the recognition model in this paper achieves a good balance between detection speed and detection accuracy. Figure 16 shows the recognition accuracy curve for different models, from which we can see that the overall recognition accuracy of our model for the target is higher than that of other models.

### 3.2. Quality Grading Method Performance Test

**Figure 17:**
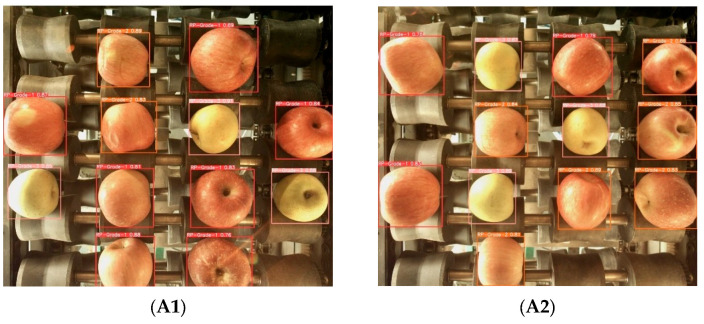

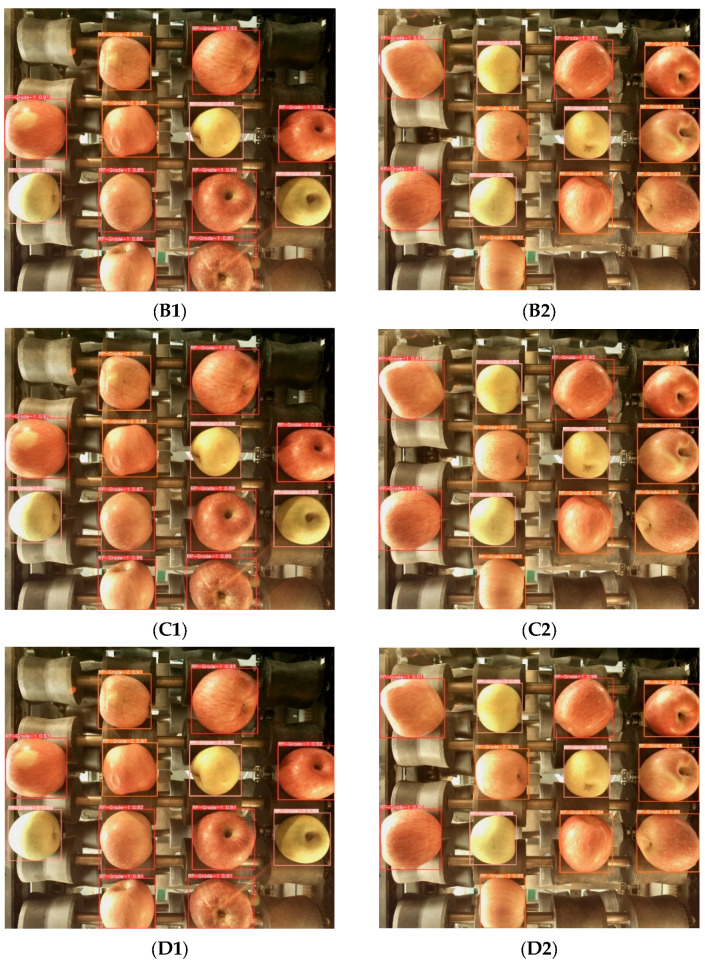
Grading results. (**A1**–**A2**) MobileNetV2; (**B1**–**B2**) ResNet18; (**C1**–**C2**) YOLO7; (**D1**–**D2**) Proposed method.

The excellent ability of the Swin Transformer to extract image features makes it perform well in multiple downstream tasks of visual detection. However, in the apple grading task with high requirements for detection speed and detection accuracy, its performance needs to be further verified by experiments. We chose MobileNetV2 [26], ResNet18 [27] and YOLOv7 [28] network models for experimental comparison. Figure 17 and Table 4 show the detection effect and performance data of different models in the apple quality grading task.

After processing the multi-dimensional apple surface information, the apple with defects detected on the surface will be directly judged as a third-grade fruit, and the judgment results obtained by the subsequent quality grading model will not be received by the grading executive agency. If an apple with fruit stems in the perspective is detected, the judgment results of the subsequent quality grading model for this perspective will not be saved. The detection results for apple images without fruit stems in the view will be saved. Finally, all of the saved detection results for apples are weighted to obtain the final grading result. As can be seen from the detection results in Figure 17, the characteristics of the third-grade fruit were quite different from those of the second-grade fruit and the first-grade fruit, and all of these algorithms could accurately identify them. However, for the detection of the first-grade fruit and the second-grade fruit with similar surface characteristics, the performance of these models was very different. The performance of the MobileNetV2 model was poor in the detection of first-grade fruit and second-level fruit, and the detection accuracy was about 80%. The model with ResNet18 as the feature extraction backbone had improved performance in the detection of second-grade fruit, but its detection accuracy for first-grade fruit was still not high enough. Although the YOLOv7 model performed well in different levels of apple detection, it can be seen from Table 4 that the detection model in this paper had obvious advantages in detection accuracy. Compared with the detection model in this paper, the number of model parameters of the YOLOv7 model was increased by 33.13 M [29], and the detection speed was reduced by 59.3%. It cannot meet the demands of the apple quality grading pipeline in terms of detection rate. In summary, the proposed method is more suitable for application in apple grading tasks with high requirements for detection rate and accuracy.

### 3.3. Experimental Results of Apple Grading

**Figure 18:**
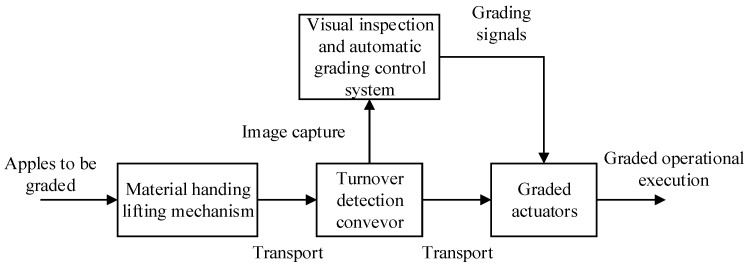
Workflow diagram for automatic apple grading.

This paper experimentally verified a four-lane apple grading machine, and its workflow is shown in Figure 18. When the apple grading device is started, the apple is lifted to the flipping conveyor through the material feeding lifting mechanism. The flipping conveyor uses a pair of double conical roller brackets to flip the apple. The grading execution device receives the grading results after the grading model has classified the apples [30]. According to the grading results evaluated by the grading control system, the grading actuator automatically puts the apple into the corresponding storage bin when it reaches the corresponding grading area.

As samples, 300 apples of every quality grade were chosen at random and randomly run through the grading machine, and the surface defects, fruit stem recognition accuracy and apple grading accuracy of each grade were recorded. The experimental results are shown in Table 5 and Table 6.

The experimental results show that the detection speed for surface defects and fruit stems reached 32 frames per second, which can quickly complete the processing of multi-dimensional information. The surface defect recognition accuracy was high, and only one defect sample was not correctly identified. For the identification and detection of fruit stems, the incorrectly identified fruit stems were all in the edge area of the apple image, so the detection was difficult. However, all of the fruit stem information in the normal region of the apple image was correctly recognized. For the apple grading experiment, the first-grade fruit and second-grade fruit were similar in shape and color, so the grading was difficult, and there were some errors in the grading results. The grading accuracy for the grade-one apples was 93.27%, the grading accuracy for the grade-two apples was the lowest, and the classification accuracy for the grade-three apples was the highest. Only one grade-three fruit was incorrectly identified because of a surface defect identification error; the average classification accuracy was 94%, and the average classification speed was 4 fruits/s. The efficiency and accuracy of quality grading meet the requirements of automatic grading. This experiment also tested the grading effectiveness of the grading model under different speeds of the flip conveyor. Taking the detection of four apples per second as the base speed (in other words, the time for an apple to pass through the detection area was about two seconds), the grading accuracy was 94% at this time. When the conveyor speed was reduced by 25%, the detection accuracy did not change significantly. When the delivery speed was reduced by 50%, the detection accuracy reached 95.6%. Although the accuracy was improved, too much detection speed was sacrificed. When the transmission speed was increased by 25%, the captured images were prone to motion blur, resulting in a decrease of 4.4% in the classification accuracy of the model. Therefore, it can be inferred that the proposed method can achieve the best balance between detection rate and accuracy at the rate of detecting four apples per second.

In order to further verify the superiority of the proposed method and compare it with other, recent apple classification methods, the comparative results are shown in Table 7. Xu [8] improved the YOLOv5s model by using an SE attention module and directly graded the quality of real-time apple images. Although multiple apples could be synchronously graded, interference information from apple images was not filtered. As a result, the grading accuracy for grade-two fruit was only 88%, which further verified that the multi-dimensional view information processing method designed in this paper can effectively improve the accuracy of quality grading. Shi used bidirectional LSTM to build a spatial feature aggregation module to integrate multidimensional information on apples. Their research could effectively improve the accuracy of apple classification, but their classification model was complex, and the detection rate could only reach 10 frames/s. Multidimensional information on apples was collected by three cameras fixed at different locations. In addition, only one apple could be collected each time, so the detection efficiency was too low and the cost was high, which was not suitable for actual production line operation.

## 4. Conclusions

In order to solve the existing problems in the synchronous quality grading task for multiple apples, this paper designed an apple quality grading method based on multi-dimensional view information processing and conducted experimental verification on a four-lane apple grading machine. The main conclusions of this paper are as follows.

(1) A multi-dimensional view information processing method was designed to solve the problem of easy access to image noise and interference information during quality grading and enable the grading model obtain more complete apple surface information. Firstly, the Retinex image enhancement algorithm was used to reduce the negative impact of uneven illumination and apple surface reflection on quality grading. Then, the detection of apple surface defects and the identification and screening of fruit stem and calyx information were simultaneously completed by improving the YOLOv5s model, and the ordinary convolution in the backbone of YOLOv5s was replaced by ODConv convolution to enhance the network’s ability to extract apple surface features. Compared with the baseline model, the recognition accuracy of YOLOv5s was improved by 2.24%, and the light weight of the neck part of the model was improved, GSConv lightweight convolution was used to replace ordinary convolution to receive feature mappings from the model backbone. After GSConv convolution, the VoVGSCSP lightweight backbone was used to complete the up-sampling and down-sampling of image features and reduce information processing time. Compared with the baseline model, the detection speed was improved by 23%, which meets the requirements of apple quality grading for detection efficiency.

(2) In order to improve the classification accuracy of the quality classification model, this paper adopted the Swin Transformer module to improve the third and fourth stages of the YOLOv5s backbone, so as to improve the comprehensive discrimination ability of the classification model for multiple features on apple surfaces. SwinT divided the image information into windows and then completed the information processing separately, which can effectively reduce the computational complexity and enhance the nonlinear expression ability of the model without increasing the computational cost too much. In the comparison experiment, the detection accuracy of the method in this paper was 94.46%, and the detection speed was 32 frames/s. The detection speed and accuracy were better compared to other classification algorithms.

In order to further improve grading accuracy and meet the needs of the automated grading pipeline for detection efficiency, future work will not be limited solely to improvement of the YOLO series model, but will also select a variety of baseline models for experimental comparison to verify which model performs better and then further improve it. The improved model is encapsulated as an application that can be easily ported to various grading devices to help increase the automation of apple grading in the market, promote the commercialization of apples, constantly improve their market competitiveness, and create greater economic benefits.

## Figures and Tables

**Table 1:** Standard for grading apple quality.

Characteristic	Grade-1	Grade-2	Grade-3
Color	Bright red, thick red coloring area ≥80%	Bright red, thick red coloring area ≥55%	Bright red, thick red coloring area <55%
Shape	Shape index ≥0.85	Shape index ≥0.80	Shape index ≥0.75
Diameter	≥70 mm	65 mm~70 mm	60 mm~65 mm
Surface defect	No flaw	No flaw	Area not exceeding 3 square centimeters

**Table 2:** Ablation experiment.

YOLOv5s	Retinex	ODConv	Slim-Neck	AP (%)	FPS (Frames/s)	Params (Million)
√				89.67	26	7.40
√	√			94.32	26	7.51
√	√	√		96.98	23	9.32
√	√	√	√	96.56	32	6.78

**Table 3:** Test results of different models.

Method	F1 (%)	Precision (%)	Recall (%)	FPS (Frames/s)	Params (Million)
YOLOX-Tiny	92.32	92.56	92.03	22	5.04
MobileNetV2	93.87	93.11	93.32	25	6.79
ShuffV2-YOLOX	94.22	94.65	94.13	34	6.65
Proposed method	96.43	96.56	96.39	32	6.78

**Table 4:** Performance parameters of different models in hierarchical tasks.

Method	F1 (%)	Precision (%)	Recall (%)	FPS (Frames/s)	Params (Million)
MobileNetV2	90.26	90.46	90.02	27	5.04
ResNet18	92.78	92.21	92.31	28	7.14
YOLOv7	93.32	93.74	93.23	19	36.91
Proposed method	94.13	94.46	94.09	32	3.78

**Table 5:** Stem and defect experimental data.

Apple Features	Total Number of Apple Samples	Unrecognized Features Number	Detection Speed (Frames/s)
Flaw	300	1	32
Stem	300	5	32

**Table 6:** Grading experimental data.

Grade	Number of Samples	Number of Correct Grades	Accuracy Rate (%)	Detection Time (Fruits/s)
Grade-1	119	111	93.27	4
Grade-2	105	96	91.42	4
Grade-3	76	75	98.68	4
Comprehensive	300	282	94	4

**Table 7:** Comparison with existing apple grading methods.

Grading Method	Accuracy Rate (%)	Detection Speed (Frames/s)	Detection Time (Fruits/s)
Hamza [31]	87.42	14	1
Xu [8]	88	16	4
Shi [6]	99.23	10	1
Proposed method	94.46	32	4

## Data Availability

The data presented in this study are available on request from the corresponding author.
